# Comparative Analysis of Soil Microbiome Profiles in the Companion Planting of White Clover and Orchard Grass Using 16S rRNA Gene Sequencing Data

**DOI:** 10.3389/fpls.2020.538311

**Published:** 2020-09-18

**Authors:** Lijuan Chen, Daojie Li, Ye Shao, Jannati Adni, Hui Wang, Yuqing Liu, Yunhua Zhang

**Affiliations:** ^1^ College of Animal Science and Technology, Anhui Agricultural University, Hefei, China; ^2^ School of Medicine, Huaqiao University, Quanzhou, China; ^3^ School of Resources and Environment, Anhui Agricultural University, Hefei, China

**Keywords:** companion planting, 16s RNA gene sequencing, microbiome, Clusters of Orthologous Groups (COGs), operational taxonomic unit classification, multiple variables analysis, machine learning models

## Abstract

Companion planting is one of the most common and effective planting methods in modern agriculture. White clover (*Trifolium repens* L.) and orchard grass (*Dactylis glomerata* L.) are two typical pastures planted together to promote each other’s growth. However, the detailed biological foundations of companion planting remain unclear. In this study, we screened typical microbiome profiles under separate and combination planting conditions using 16s RNA gene sequencing techniques. We identified the typical distinctive microorganism subtypes based on the microbiome profiles and recognized the enriched functions of top abundant microorganisms in soil using different planting strategies with the help of Kyoto Encyclopedia of Genes and Genomes and Clusters of Orthologous Groups annotation. This analysis confirmed that the optimal microorganisms and screened functional annotations are correlated with nitrogen fixation; thus, companion planting may improve the yield and efficacy of plants by improving the efficiency of nitrogen fixation.

## Introduction

Companion planting is another typical agricultural pattern partially associated with organisms (Finch et al., 2003; Parker et al., 2013). Companion planting is a method of planting different kinds of plants at the same time in proximity (Finch et al., 2003; Szafirowska and Kolosowski, 2008). Companion planting can help in pest control (Parker et al., 2013), pollination (Hagiwara et al., 1995; Moeller, 2004), nutrition supply optimization (Mengel, 2001; George et al., 2013), and the maximization of the use of space (Bomford, 2004). For instance, soybeans can provide nitrogen with the help of certain microorganisms in soil (Oyekanmi et al., 2007; Chen et al., 2012). Soybeans can remodel soil microbiome and provide more nitrogen nutrition in proximity for plant growth (Chen et al., 2012). Therefore, the companion planting of soybean and *Medicago sativa* may help improve the production of both plants through the modification of soil microorganisms (Plaza et al., 2003).

White clover (*Trifolium repens* L.) is a typical agricultural plant from the bean family Fabaceae (Davidson, 1969). The companion planting of white clover and grain crops or pasture grasses has been widely applied in poor soils to provide green cover (Sood et al., 2018). Orchard grass (*Dactylis glomerata* L.) also known as cat grass is a famous kind of pasture with high yield and great drought tolerance (Bybee-Finley and Ryan, 2018). White clover and orchard grass are two typical and traditional model plants for companion planting, which greatly improve their efficacy and yield rate. Early in 1962, a Canadian journal has reported the effects of companion planting on oats and confirmed that clover has equal to or greater yields when planted with the crop and orchard grass (Davis, 1962). However, the detailed mechanisms of the interactions between white clover and orchard grass still remain unclear. More progressions have been made in companion planting with the development of modern culture and sequencing technologies in the last 5 years. The improved yields can be attributed to the enhanced efficiency of nitrogen fixation induced by companion planting (Bybee-Finley and Ryan, 2018; Chalk, 2018; Payne, 2019). The improved nitrogen formulation efficiency is induced by the remodeling of microorganisms in proximity (Moore et al., 2019). However, the detailed mechanisms of the biological basis of companion planting are still unclear and require further studies.

In this study, we focused on the companion planting of white clover and orchard grass using 16S rRNA gene sequencing techniques (Pitombo et al., 2016; Smets et al., 2016). We monitored microbiome remodeling patterns in separate and companion planting conditions. The differential and altered microbiome distribution patterns confirmed the contribution of microbiome in the companion planting of the two plants and partially revealed the potential biological foundations for companion planting at least at the microbiome level. In this study, we revealed the biological foundation of the companion planting of white clover and orchard grass at the microbiome level, constructed a general workflow to study the contributions of microbiome on companion planting, and provided a new perspective on the biological foundations of companion planting.

## Materials and Methods

### Experiment Site and Soil

Experiments were performed at the Dayangdian Experimental Station of Anhui Agricultural University (31°58′N, 117°24′E), Hefei City, Anhui Province, Southeast China. The study site is located between the Yangtze River and Huaihe River. The study area belongs to the transitional zone between the warm temperate zone and subtropical zone and has subtropical humid monsoon climate. Its annual temperature is cold in winter (8–17°C), hot in summer (21–29°C), and mild in spring and autumn, and its annual precipitation is 992 mm. The soil had the following physicochemical properties on a dry weight basis: 0.89% organic matter, 81.1 mg kg^−1^ available N, 16.3 mg kg^−1^ available P, and 100.5 mg kg^−1^ available K. In May 2019, 0.25 kg soil samples were collected in the rhizospheres of the white clover (WC) and orchard grass (OG) groups and soil samples of the companion planting of both plants (Mixed). All soil samples were preserved under the same conditions, and some fresh soil samples were further processed.

### Treatments and Field Management

To explore the effects of white clover and orchard grass on the soil microorganisms, we established research sites in October 2018 where we applied three treatments: white clover, orchard grass, and the companion planting of both plants. The area of the land used in the experiment measured 4 × 4 m^2^ in each plot. The amounts of white clover and orchard grass sowed were 10 and 20 kg ha^−1^, respectively, and the Mixed group had 7.5 kg ha^−1^ white clove and 5 kg ha^−1^ orchard grass. The soil was watered when precipitation was insufficient.

### DNA Extraction and Library Construction

Total genomic DNA was extracted using DNA Extraction Kit following the manufacturer’s instructions. The quality and quantity of DNA were verified through spectrophotometry using NanoDrop spectrophotometer and *via* agarose gel electrophoresis. The extracted DNA was diluted to a concentration of 1 ng/μl and stored at −20°C until further processing. The diluted DNA was used as template for the polymerase chain reaction (PCR) amplification of bacterial 16S rRNA genes using barcoded primers and TaKaRa Ex Taq. The V3–V4 variable regions of 16S rRNA genes were amplified with universal primers 343F and 798R for bacterial diversity analysis.

Amplicon quality was visualized through gel electrophoresis, purified with AMPure XP beads (Agencourt), amplified for another round of PCR, and purified with AMPure XP beads again. The final amplicon was quantified using Qubit dsDNA assay kit. Equal amounts of purified amplicon were pooled for subsequent sequencing.

### 16S rRNA Gene Sequencing Result Analysis

#### Quality Control for Raw Sequencing Data

The raw image data obtained from high-throughput sequencing data was transformed into the original rRNA sequence in FASTQ file format by base calling analysis (Kao et al., 2009). The data in FASTQ format were further processed to remove the sequences with low quality and abnormal length using Trimmomatic software (Bolger et al., 2014). We also used UCHIME software to remove chimera in the raw FASTQ file to provide clean data for further analyses (Rognes et al., 2016). The distribution of sequence length after data cleaning is shown in the histogram and density map in **Figure S1**. Nearly all the reads distributed were within the length range of 400–450 bp with quite high quality; thus, our quality control procedure was efficient, and the clean data were eligible for further analysis.

#### Operational Taxonomic Unit Classification

We used Vsearch software to classify the high-quality sequence of the valid tags obtained by quality control according to 97% similarity. The most abundant sequence in each OTU was chosen as the representative sequence. We applied the Ribosomal Database Project classifier, the Naive Bayesian classification algorithm (Wang et al., 2007), to further align and annotate the representative sequences against the annotation database for the species information of each OTU. We further summarized the distribution of OTUs in different samples and the annotations of tags and OTUs based on the species results to show the general species distribution pattern of different samples. We used flower plot to show the numbers of shared and unique OTUs among different samples (**Figure S2**). Standardized the original data in OTU table file (the form of biom), and then the predicted functional (KEGG/COG) results were obtained by mapping the standardized data with the species functional genes from the online sequenced genome.

#### Analysis of Biome Structure From Soil in Proximity

Community structure or “biological community” refers to all the organisms that have a direct or indirect relationship with each other. Various groups in a microbial community interact with each other and can coexist in a regular manner but have their own distinct types of nutrition and metabolism. In this study, we summarized the composition of microbiome communities. We performed ternary plot analysis (Graffelman and Camarena, 2008) to compare and analyze the species composition of the three groups according to the classification results.

#### Alpha Diversity Analysis

Alpha diversity, which reflects the diversity of species in shared habitats, was calculated to present the species diversity in each sample (Huttenhower et al., 2012). Microhabitats have been tested for differences in estimated abundances with the Kruskal–Wallis significance test for all pairwise combinations. We measured the number of species and the uniformity of species abundance used the indexes of Shannon and Chao based on a rarefied (18,860 reads) dataset (**Figure S1**) to quantitatively evaluate species diversity.

#### Beta Diversity Analysis

Beta diversity is the diversity of the relationships between organisms and environment in proximity (Kurilshikov et al., 2017). Similar with the alpha diversity analysis, we also used some quantitative parameters to evaluate the differential beta diversity patterns in different groups. In this study, we used principal co-ordinate analysis (PCoA) based on Bray Curtis to reveal the beta diversity among different groups.

#### Multiple Variable Analysis of Soil Microbiome

We used OTU and species data to identify the specific species that have statistically significant difference in abundances. We used ANOVA to identify the most substantial differentially existing species among the three groups (Rojewski et al., 2012).

#### Correlation Analysis and Prediction Using Machine Learning Models

We analyzed the correlations of different species and their contribution on the distinction of different groups using correlation analysis and machine learning methods. We also applied random forest apart from direct correlation analysis for further analysis. Random forest is the machine learning algorithm first proposed by Leo Breiman and Adele Cutler in 2001 (Breiman, 2001). Random forest is regarded as an integrated learning method with multiple decision trees. The output classification result is the result of “voting” by each decision tree. The classification results of random forests have high accuracy and do not need to “cut branches” to reduce overfitting because each tree uses random variables and random sampling methods in the construction process (Breiman, 2001; Segal, 2004). We used the proper R package (random forest) to perform the random forest algorithm (Segal, 2004).

#### Phylogenetic Investigation of Communities by Reconstruction of Unobserved States Analysis

PICRUSt functional predictive analysis is based on 16S rRNA gene sequencing data and annotated by Greengenes database (DeSantis et al., 2006). The PICRUSt software (Langille et al., 2013) is widely used to analyze the functional genetic composition of identified microorganism to reveal the functional diversity between different samples or groups. In this study, we applied PICRUSt analysis workflow (Langille et al., 2013) to reveal the functional distribution patterns of the different samples and groups.

## Results

### Effect of Companion Planting on Microbiome Community Structure and Abundance

**Figure 1:**
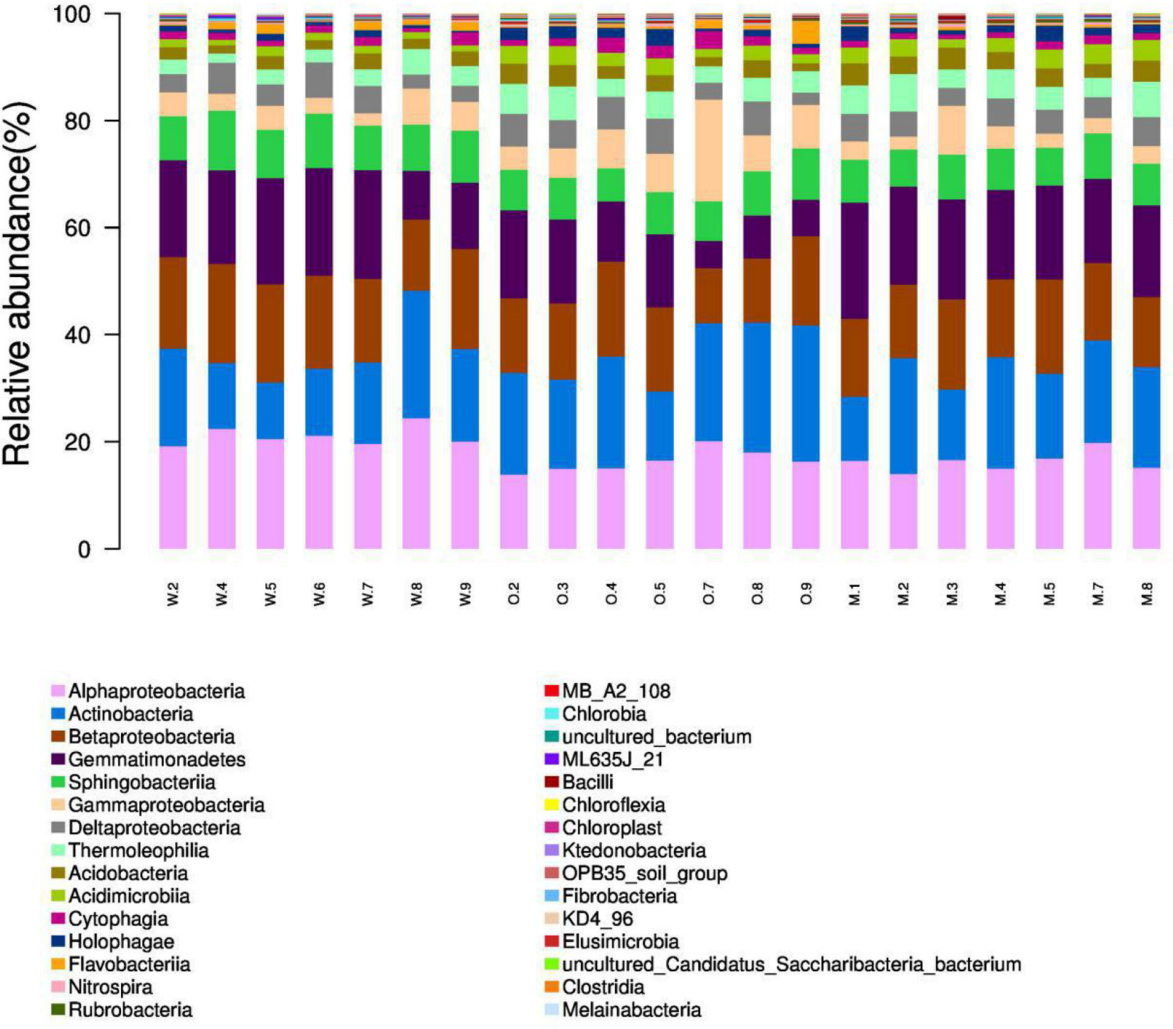
Microbiome composition of the microorganisms with the top 30 abundances at the class level. We calculated the relative abundances (%) of the top 30 most abundant microorganisms at the class level. The proportion of top classes, such as Alphaproteobacteria, Actinobacteria, Betaproteobacteria, Gemmatimonadetes, was great in most samples regardless of groups and reflects the background microbiome pattern in the proximity. (Note: W2, W4–9 are WC samples; O2–5, O7–9 are OG samples; M1–5, M7–8 are Mixed samples).

**Figure 2:**
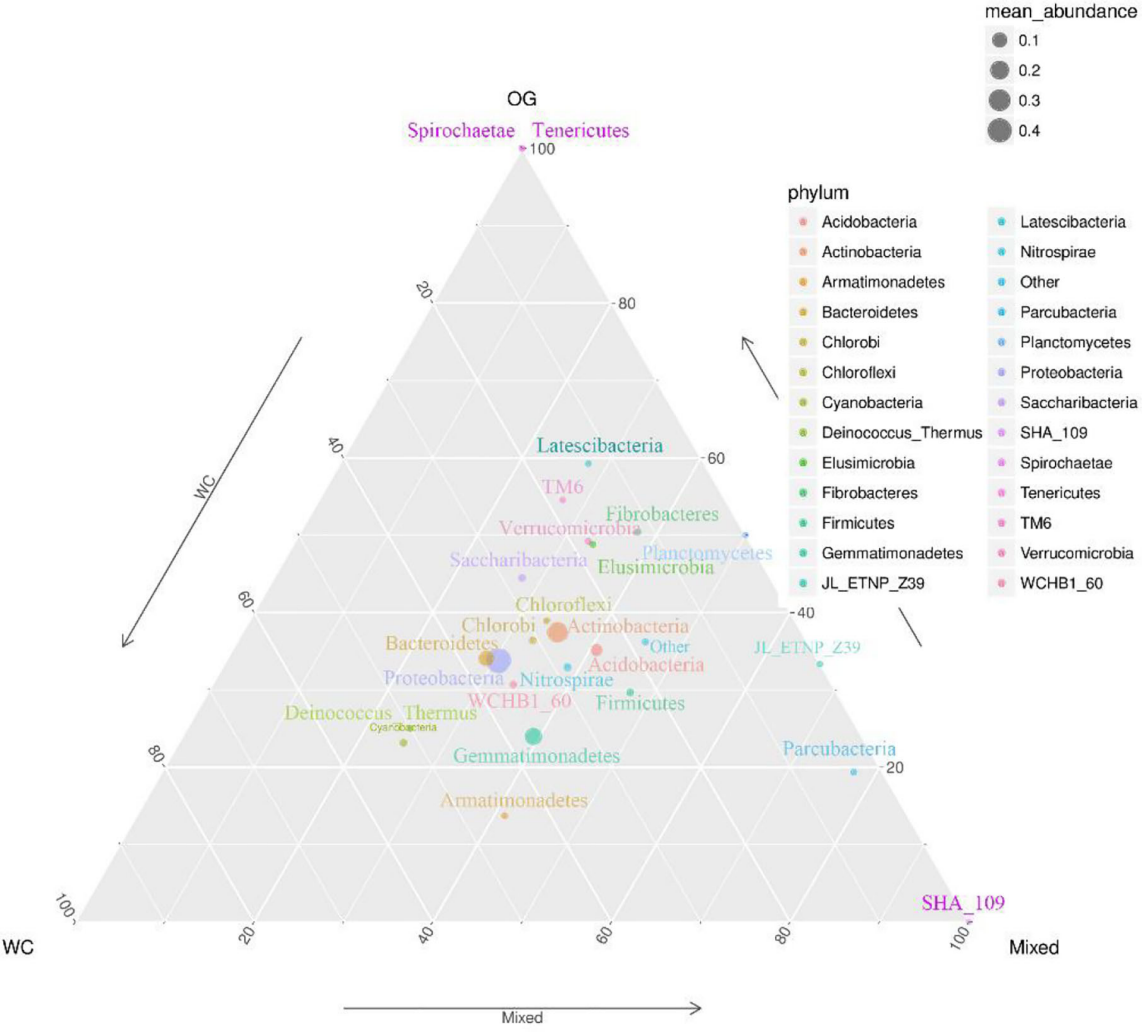
Ternary plot of the contribution and relationship of different species in different groups. The ternary plot shows the unique species distribution patterns and abundances of the three groups.

We summarized the composition of the microbiome community at the class level according to the microorganisms with the top 30 abundances (**Figure 1**, **Table S1**). According to the result, specific classes, such as Alphaproteobacteria, Actinobacteria, Betaproteobacteria, Gemmatimonadetes, had top abundances in nearly every sample and reflected the background microbiome distribution pattern in proximity. However, some specific classes, such as Gemmatimonadetes, had relatively higher abundance in the Mixed group compared with the OG group and indicated the potential microbiome remodeling effects of companion planting. However, the differential distribution patterns of the microbiomes of the three different groups were not clear. Therefore, we also used the ternary plot to reveal the contribution and relationship of different Phylum in different groups (**Figure 2**). According to **Figure 2**, Tenericutes and Spirochaetae were found in the specific distribution pattern of the OG group. This result indicated that these two microbiomes may be unique under the OG planting pattern and verified that companion planting affects the microbiome distribution pattern in proximity.

### Effect of Companion Planting on Alpha Diversity

We used the boxplot to show the alpha diversity using Shannon and Chao1 parameters (**Figures S4**, **S5**). Results showed that the OG and Mixed groups had remarkably higher Chao1 (community richness) index values compared with the WC group (*P* = 0.0103; *P* = 0.0029). However, no significant difference was shown in Shannon index among there groups (*P* = 0.0545). Chao1 index describes and evaluates the number of species. A higher Chao1 index indicates a higher number of species in the sample. The Mixed group had the most diverse microbiome among the groups and had similar species abundance as the OG group (*P* = 0.713). The OG and Mixed groups had higher Chao1 index than the WC group. The differences of the three groups in Chao1 index indicated that their microbial diversity and microbiome abundance are quite different from each other.

### Effect of Companion Planting on Beta Diversity

**Figure 3:**
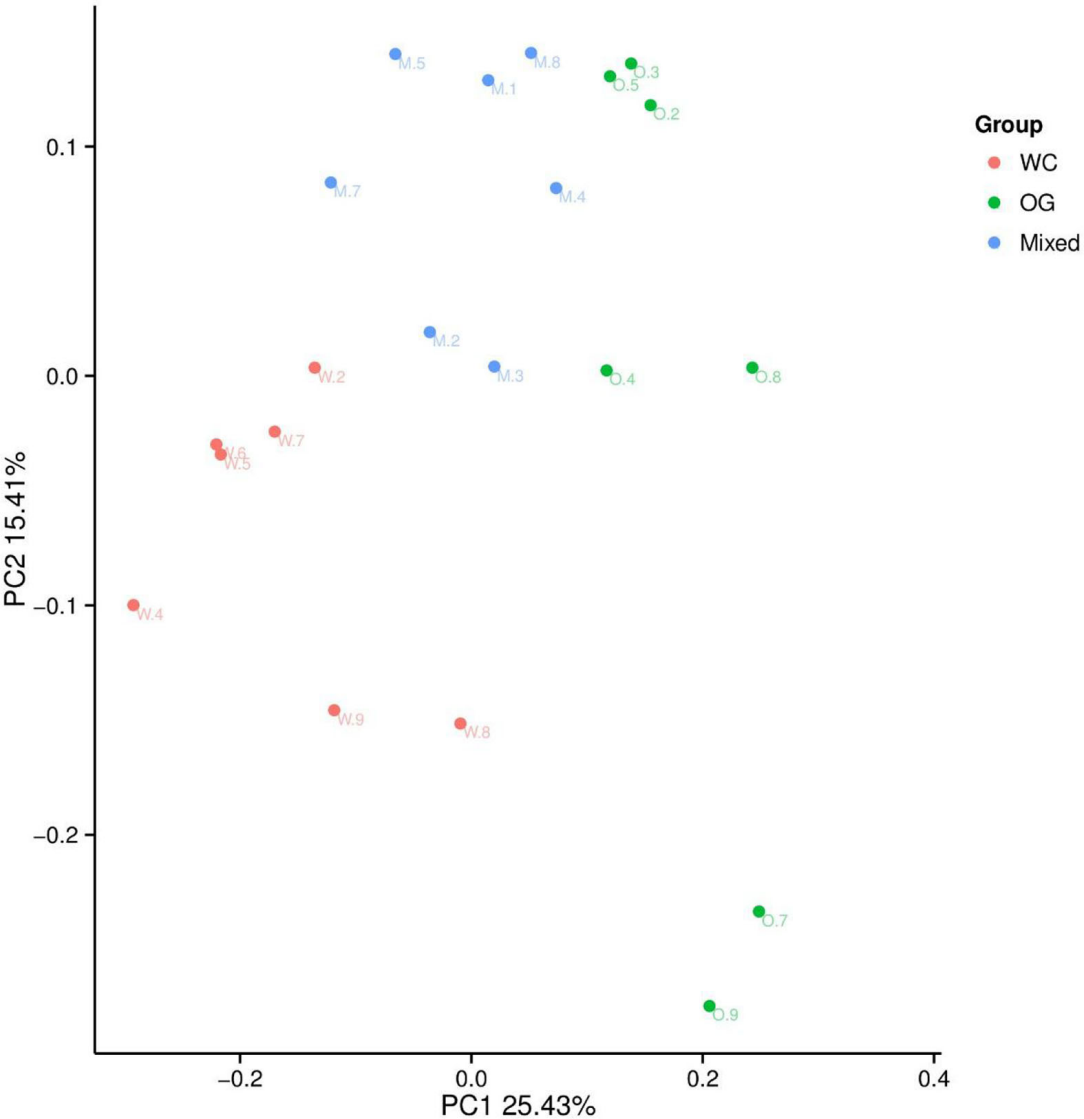
PCoA analysis of the OTU composition differences of the three groups. The microbiome distribution and diversity of three groups were separated into different parts.

We used Bray Curtis distance to evaluate the relationships between different samples and groups. The results were similar to those of the alpha diversity analysis. The three groups were divided into different parts. The mixed groups were distributed between WC and OG. However, the mixed groups were closer OG than WC, which indicated that the microbial community of OG played the main function in companion planting. Similar results were also shown by the PCoA results (**Figure 3**).

### Effect of Correlation Analysis on Specific Contribution on Genus Level

**Figure 4:**
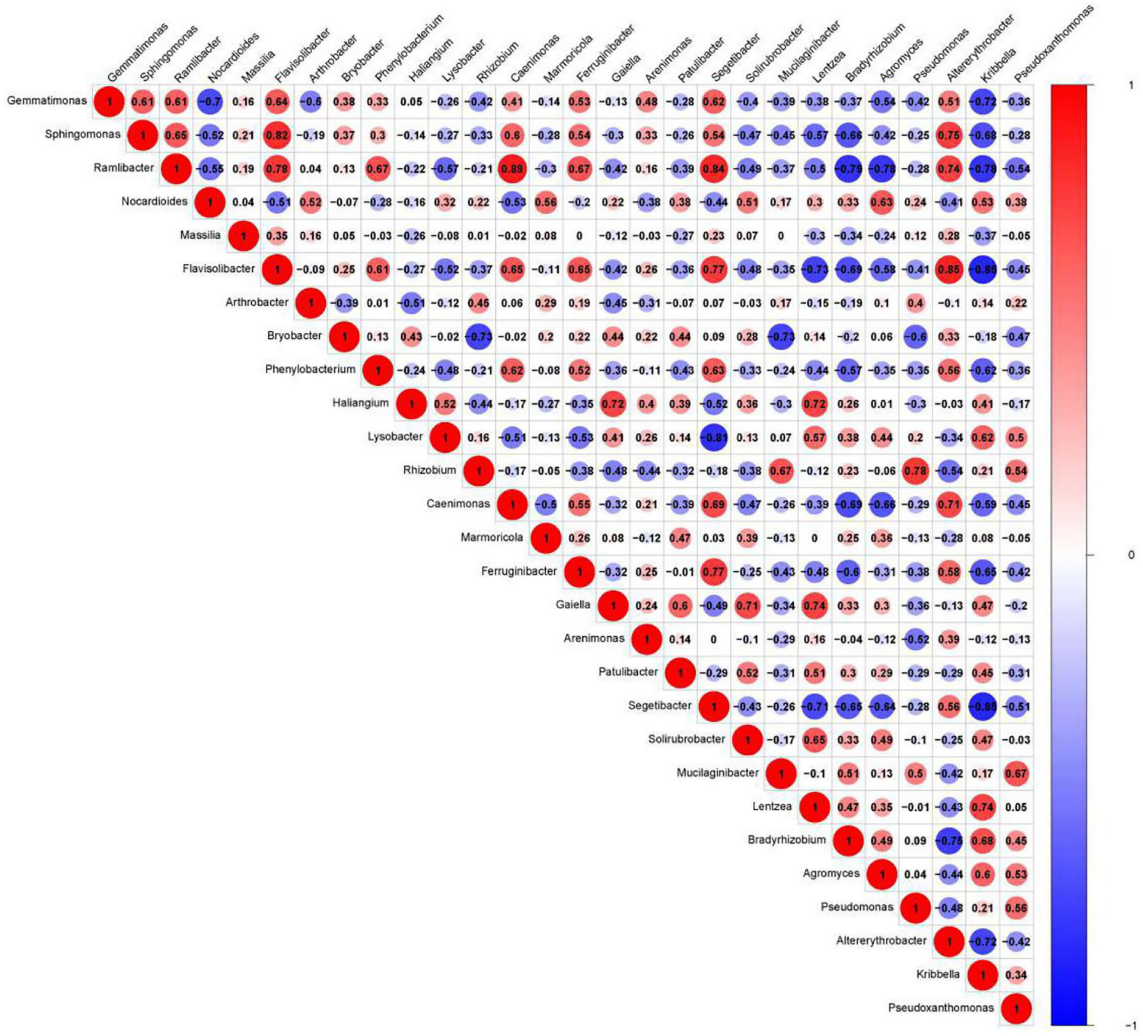
Correlation plot of the top 30 genera with the highest abundance. The plot shows the inner correlation between different species (FDR < 0.1). Red indicates negative correlations, and blue indicates positive correlations at the abundance level. Size of the circle indicates absolute strength of correlation.

**Figure 5:**
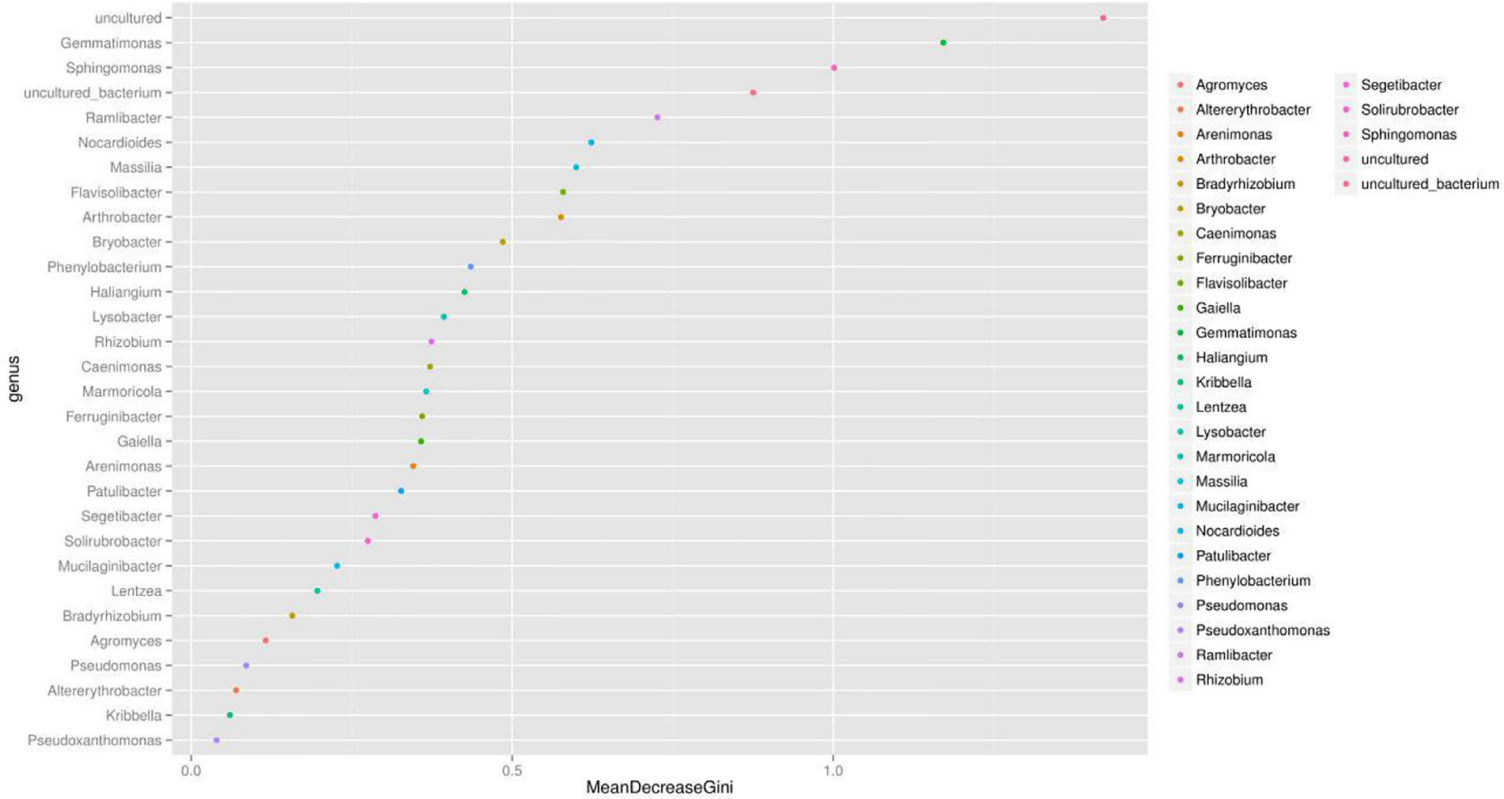
Random forest evaluation of the top 30 genera for the distinction of different groups. We used random forest method and evaluated the importance of these features using the mean decrease grid. The point map of genus importance (variable) is shown. The abscissa is the measure of importance, and the ordinate is the name of the genus sorted by importance. Standardized importance values are used by default.

We presented the correlation analysis results using a correlation plot (**Figure 4**) to show the inner relationship among the top 30 genera with the highest abundance. We also showed the specific contribution of each genus through a random forest in **Figure 5** based on the classification of the three subgroups. We ranked the contribution of each genus according to the mean decrease grid parameter and showed the top genera that contributed to the distinction of the three groups. Unique genera, such as *Gemmatimonas* and *Sphinomonas*, are quite important for the distinction of the three groups.

### Effect of Companion Planting on Functional Differential Enrichment

**Figure 6:**
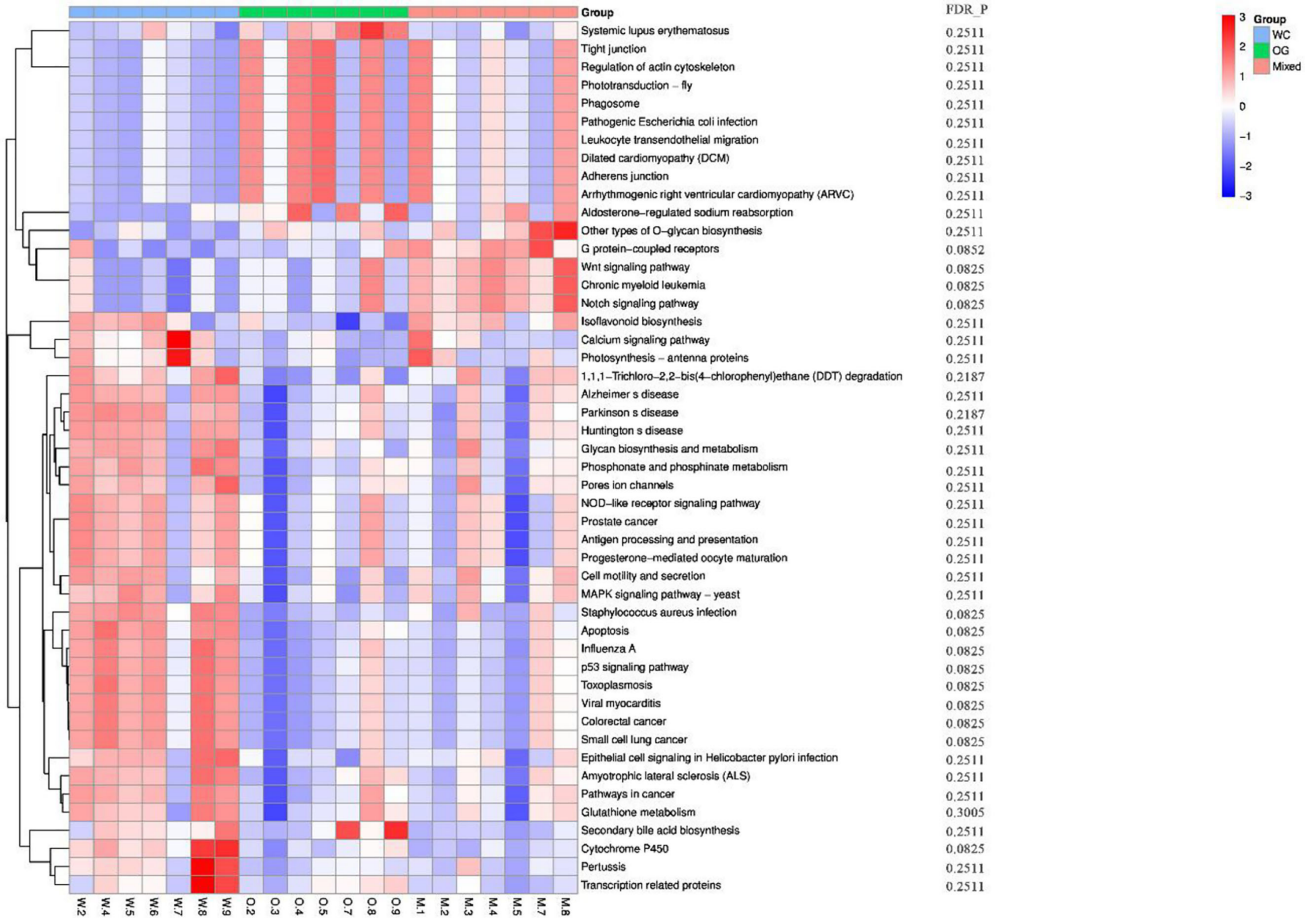
KEGG functional annotation and differential enrichment analysis of the three groups. The functional annotation and clustering of the top KEGG terms were performed using the KEGG database. The samples from different groups are difficult to distinguish using KEGG terms.

**Figure 7:**
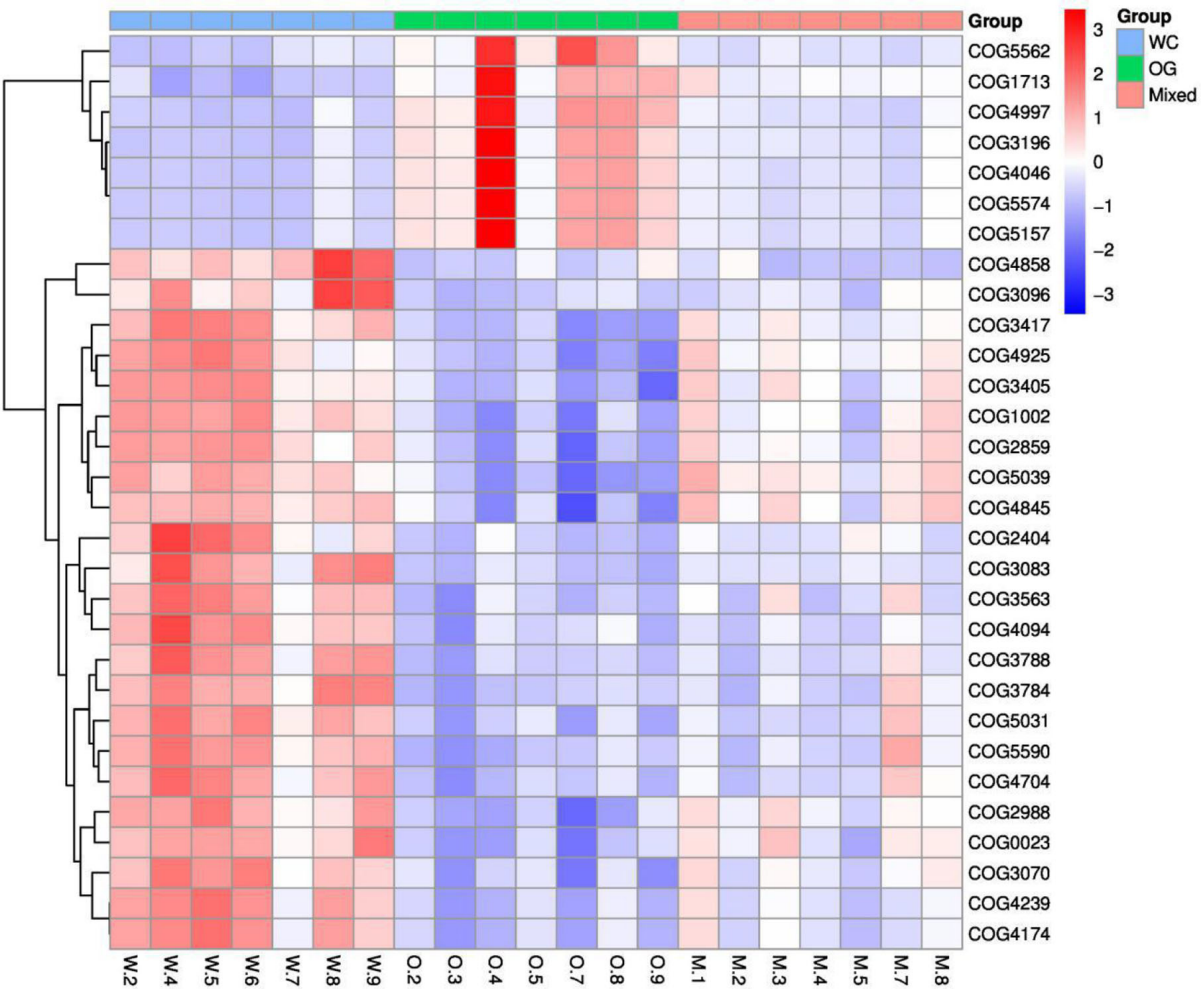
COG functional annotation and differential enrichment analysis of the three groups. The samples were screened for the top enrichment functions using their annotation from the COG database (FDR = 0.0907). The samples from the WC group can be easily distinguished from the OG and Mixed groups. The different functional distributions of the different groups are shown.

We used Kyoto Encyclopedia of Genes and Genomes (KEGG) (Kanehisa et al., 2002; Aoki and Kanehisa, 2005) and Clusters of Orthologous Groups (COGs) (Galperin et al., 2017) functional annotation and prediction to show the distribution of functional clusters among different samples and groups. We could not distinguish the samples from the three groups using KEGG enrichment analysis (**Figure 6**). By contrast, we were able to distinguish the WC group from the OG and Mixed groups using COG annotation and enrichment analysis (**Figure 7**). This finding is similar with previous functional analysis, which indicated that the effect of planting only white clover on the microbiome is quite different from those of planting only orchard grass and companion planting.

## Discussion

### Effect of Companion Planting on Soil Bacteria Community Structure and Diversity

The samples from different groups have different biome structures. **Figure 1** demonstrates that most of the samples from three groups have similar microbiome compositions, but relatively abundance differ among three groups at the OTU level (**Table S1**). Similar results have been reported in previous studies on soil microbiome (Gołębiewski et al., 2014; Samad et al., 2017). These results confirmed the complexity of soil at the microbiome level. We also identified some unique distribution patterns at the class level. Alphaproteobacteria, Actinobacteria, Betaproteobacteria, Gemmatimonadetes were detected in almost all the samples and reflect the general soil background in the proximity. Alphaproteobacteria, Actinobacteria, Betaproteobacteria, Gemmatimonadetes are widely detected in farmlands and pastures all over the world (Aguilar et al., 2004; Rosenblueth and Martínez-Romero, 2004; Reina-Bueno et al., 2012).

We identified two unique species from Tenericutes and Spirochaetae that contributed to distinguishing the OG group from the other two groups. Tenericutes has been identified in regions with various kinds of grass orchards worldwide (Liu et al., 2017; Deakin et al., 2018). Spirochaetae has also been identified in regions planted with grass orchards (Brown, 1943). Here we can’t find Tenericutes and Spirochaetae in soil of WC and Mixed group. These findings may imply that some root exudates in WC inhibit the specific distribution of these microorganisms.

### Effect of Companion Planting on Bacteria Groups

Genus *Gemmatimonas* contributed the most to the distinction of the groups (**Figure 4**). *Gemmatimonas* may participate in nitrogen fixation processes and inhibit plant pathogens in the soil (Abed et al., 2010; Peng et al., 2019). Therefore, the identification of this genus may indicate differential nitrogen fixation process efficacy among different groups and indicates that improving nitrogen fixation efficacy may be one of the biological foundations of companion planting. Other bacterial genera also participate in nitrogen fixation, such as *Sphingomonas* (Xie and Yokota, 2006), *Ramlibacter* (Thanh and Diep, 2014), and *Nocardioides* (Lim et al., 2014). Differential abundance analysis showed that the nitrogen fixation-associated bacteria of the different groups were different. Therefore, nitrogen fixation is of the biological bases and microbiome effects of improving the efficacy of planting by companion planting.

### Effect of Companion on Functional Differential Enrichment

According to the COG annotation and clustering results, some COG terms have different enrichment patterns in the different groups, especially in the Mixed group. For instance, COG1713 and COG5574 were enriched in the Mixed group, and COG1713 had a high enrichment pattern in the Mixed group. According to the EggNog database (Powell et al., 2014), COG1713 describes the co-enzyme transport and metabolism processes in bacteria, such as *Treponema azotonutricium* ZAS-9. According to an independent study on the symbiotic nitrogen fixation in New Zealand (Reid and Lloyd-Jones, 2009), bacteria plays an effective role in nitrogen fixation in pasture regions. Therefore, the activation of these biological processes may contribute to the improvement of nitrogen fixation.

The other COG term, COG5574, has been supported by Wani et al. (2007). COG5574 describes the post-translational modification, protein turnover, and chaperones involved in various ion binding processes. In 2007, a systematic analysis (Wani et al., 2007) on the molecular genetics of white clover confirmed that the binding of cadmium, chromium, and copper ion is functionally related to nitrogen fixation in this plant. Therefore, the identified biological process is also functionally related to nitrogen fixation processes.

## Conclusion

We compared the microbiome distribution patterns of planting white clover and orchard grass under single planting and companion planting conditions using 16S rRNA gene sequencing techniques. The analysis results confirmed that the companion planting of white clover and orchard grass can remodel soil microbiome in proximity, especially when compared with the single planting of white clover. We identified a group of differentially distributed microorganisms, such as Gemmatimonadetes. We also identified a group of biological processes, namely, COG1713 and COG5574, using functional annotation and clustering. The screened microorganisms and functional enrichment patterns indicate the specific role of nitrogen fixation effects during companion planting. Therefore, we were able to screen the specific microbiome distribution patterns at the species and functional levels and confirm that nitrogen fixation is one of the most important biological mechanisms for companion planting.

## Data Availability Statement

The datasets generated for this study can be found in NCBI (https://www.ncbi.nlm.nih.gov/sra/PRJNA625872).

## Author Contributions

All authors contributed to the article and approved the submitted version. LC and YZ designed the study. LC and DL performed the experiments. YS, HW, and YL analyzed the results. LC wrote the manuscript.

## Funding

This work was supported by grants from the National Natural Science Foundation of China (31872418), the Natural Science Foundation of Anhui Province (1808085MC60), the Science and Technology Research Projects of Anhui Province (201904b11020043, 201904e01020014), and the National Key Research and Development Program of China (2018YFD1100104).

## Conflict of Interest

The authors declare that the research was conducted in the absence of any commercial or financial relationships that could be construed as a potential conflict of interest.

## Supplementary Material

Supplementary Figure 1 The rarefaction plots for chao1.

Supplementary Figure 2 Histogram and density map for clean tags length after data cleaning. It’s easy to figure out that after data cleaning, all the high-quality reads locate in the range between 400 bp and 450 bp, indicating that the quality control procedure is effective and such clean data is eligible for the downstream analyses.

Supplementary Figure 3 Flower plot for the OTU number distribution pattern among different groups. The numbers in core represent the common OTUs in all samples (*i.e.*, core OTUs), and the numbers on the petals represent the total OTUs of each sample minus the number of common OTUs.

Supplementary Figure 4 Box plot for the Shannon index to evaluate the alpha diversity of different groups. Here, we compared the Shannon index of all the three groups (the mixed for white clover, the OG for orchard grass and the WC for companion planting). Planting only white clover may have lower species diversity comparing to only planting orchard grass and companion planting.

Supplementary Figure 5 Box plot for the Chao (community richness) index to evaluate the alpha diversity of different groups. Here, we further compared the Chao index of all the three groups (the mixed for white clover, the OG for orchard grass and the WC for companion planting). Planting only white clover may have lower species abundancy comparing to only planting orchard grass and companion planting.

Supplementary Table 1 The relative abundance of dominant community to each group on class level (%).Values with different lowercase superscript letters in the same row indicate the existence of a significant difference (*P* < 0.05); values with the same letters indicate no significant difference (*P* > 0.05).
