# Supplementary material for: Comparative Analysis of Soil Microbiome Profiles in the Companion Planting of White Clover and Orchard Grass Using 16S rRNA Gene Sequencing Data
Source: Front Plant Sci. 2020 Sep 18;11:538311. doi: 10.3389/fpls.2020.538311 (PMC7530175; doi:10.3389/fpls.2020.538311)
Supplement: Supplementary Figure 1 — The rarefaction plots for chao1. [file Image_1.pdf]

Appendix Figure

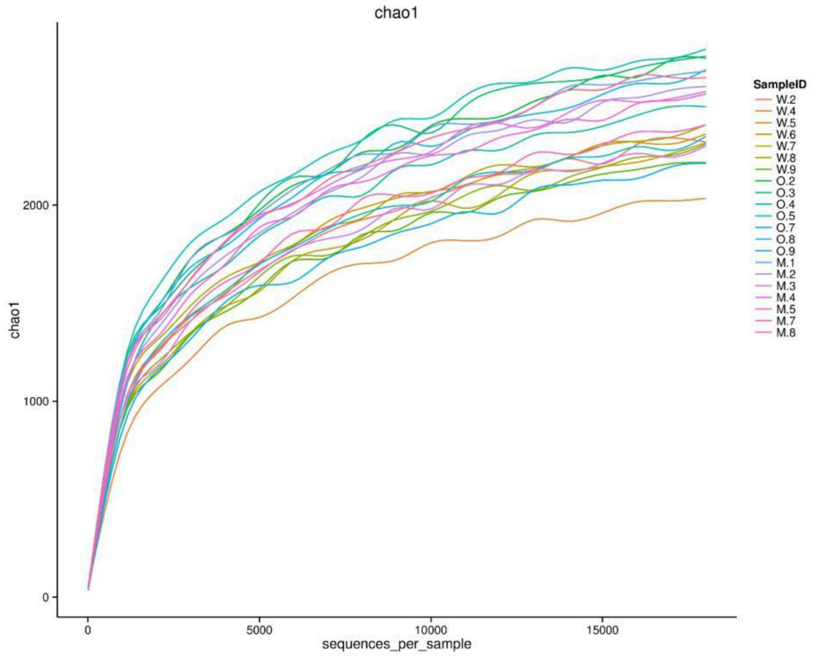

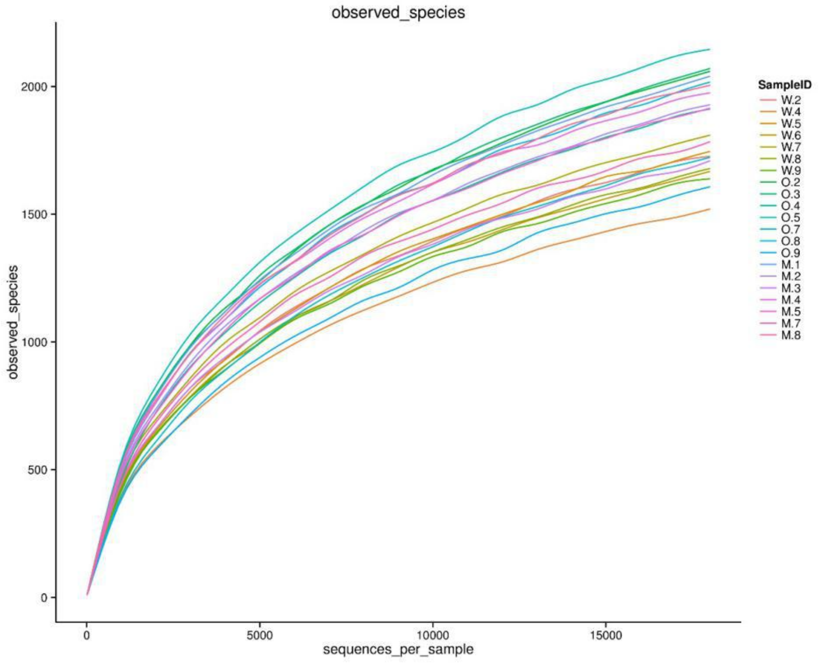

Figure S1: The rarefaction plots for chao1.

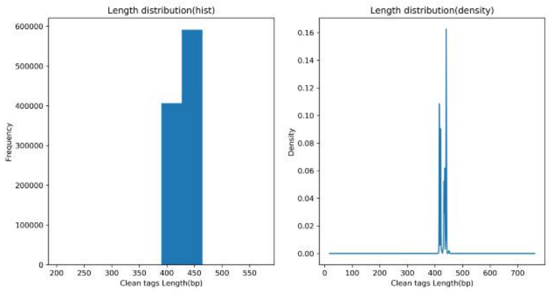

Figure S2: Histogram and density map for clean tags length after data cleaning. According to the figures, it's easy to figure out that after data cleaning, all the high-quality reads locate in the range between 400bp and 450 bp, indicating that the quality control procedure is effective and such clean data is eligible for the downstream analyses.

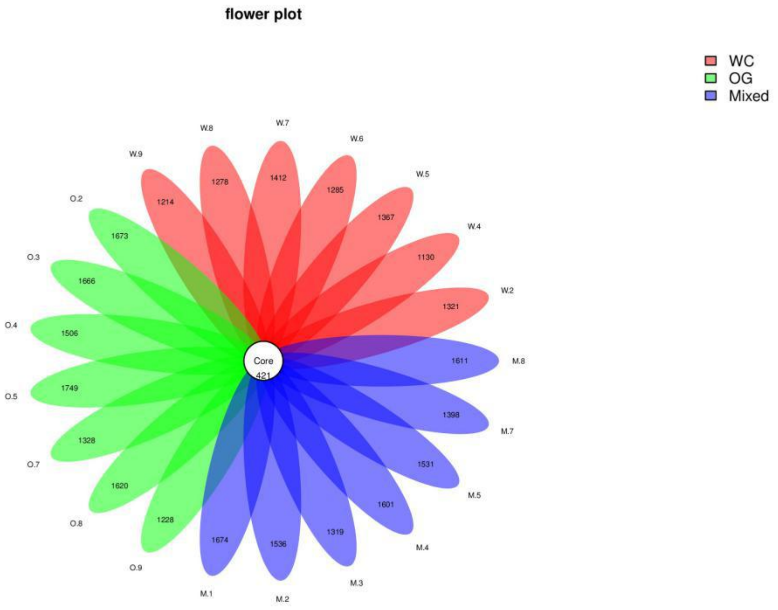

Figure S3: Flower plot for the OTU number distribution pattern among different groups. In the figure, the numbers in core represent the common OTUs in all samples (ie, core OTUs), and the numbers on the petals represent the total OTUs of each sample minus the number of common OTUs.

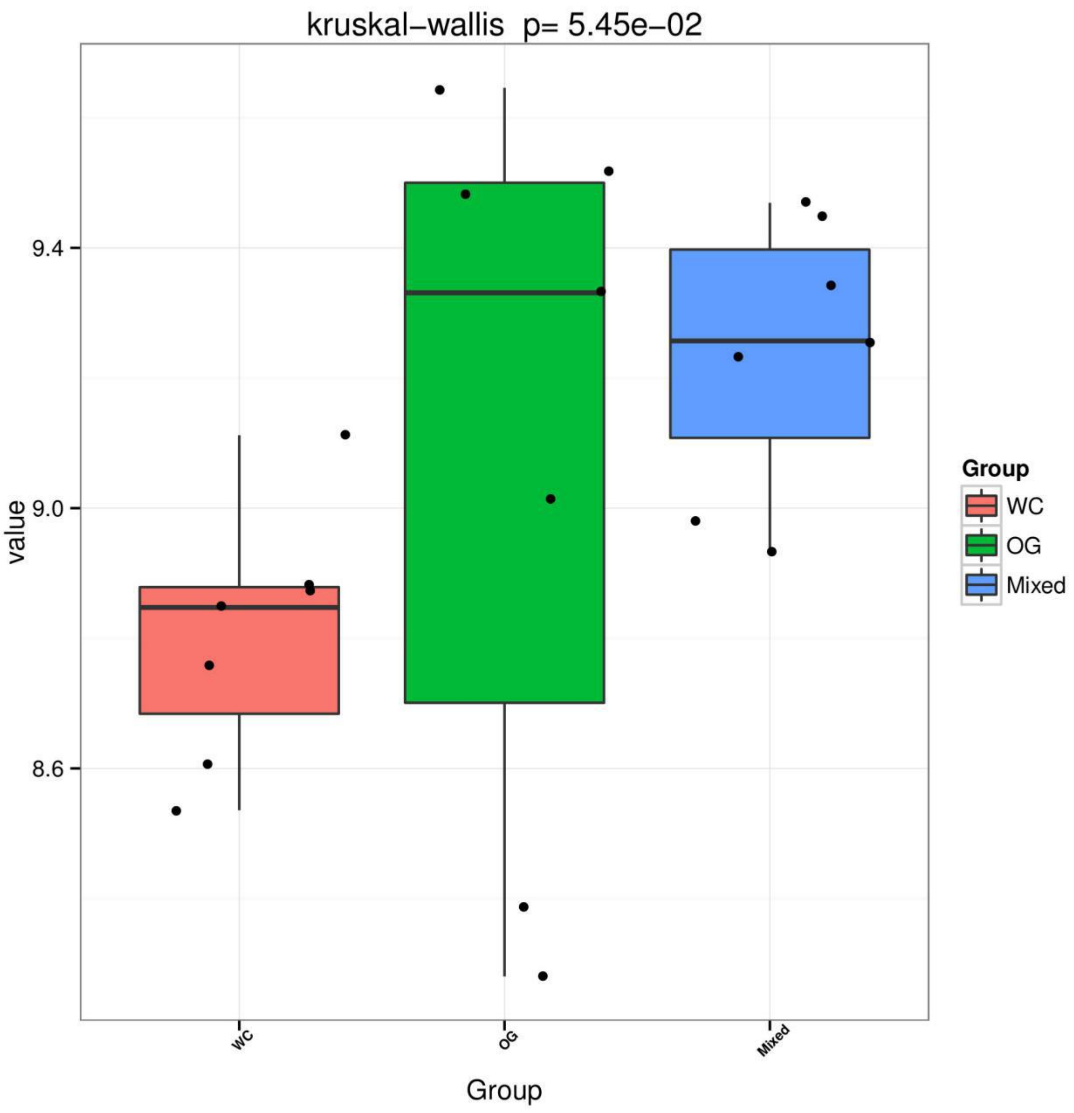

Figure S4: Box plot for the Shannon index to evaluate the alpha diversity of different groups. Here, we compared the Shannon index of all the three groups (the mixed for white clover, the OG for orchard grass and the WC for companion planting). Planting only white clover may have lower species diversity comparing to only planting orchard grass and companion planting.

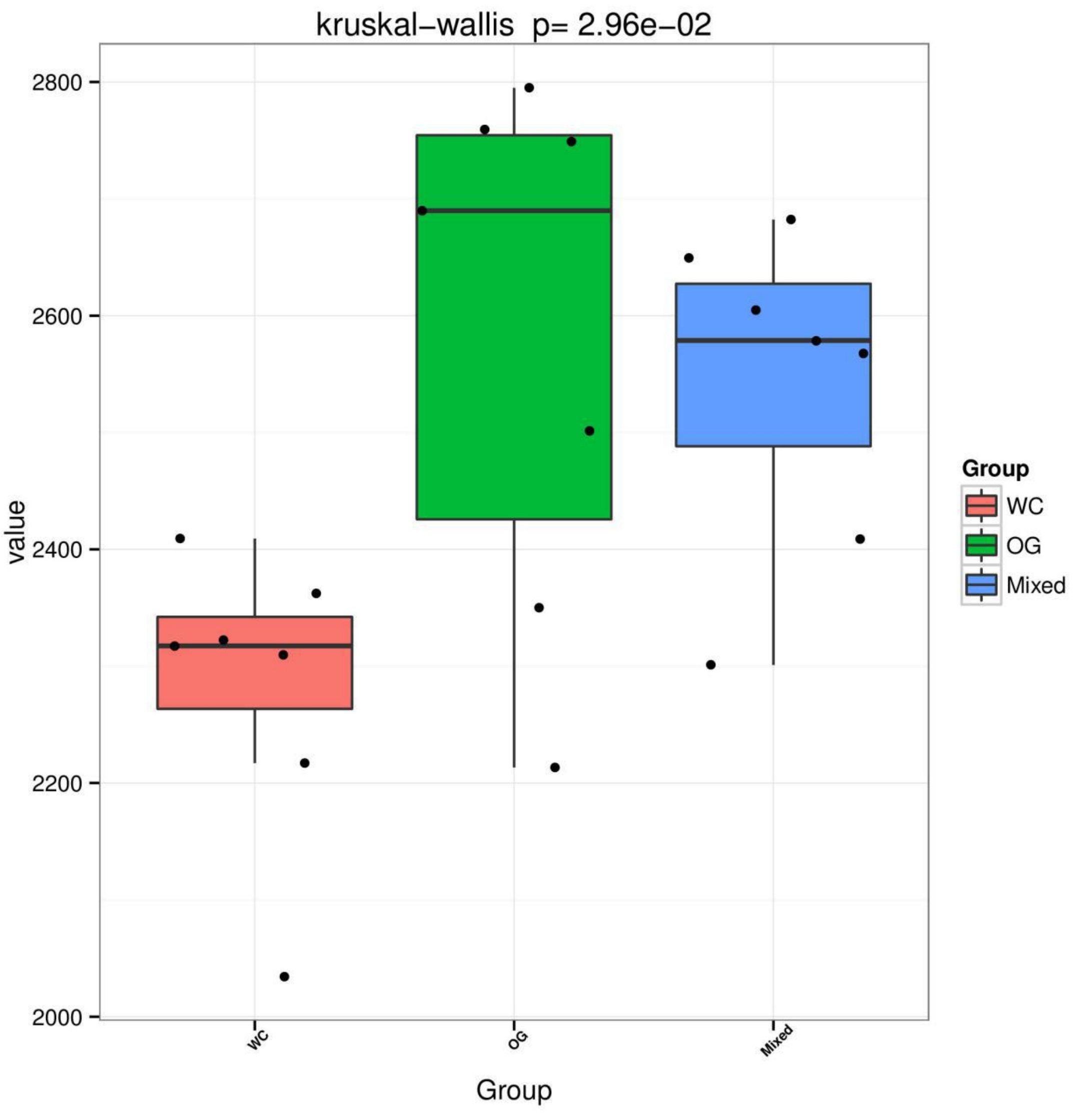

Figure S5: Box plot for the Chao1 (community richness) index to evaluate the alpha diversity of different groups. Here, we further compared the Chao1 index of all the three groups (the mixed for white clover, the OG for orchard grass and the WC for companion planting). Planting only white clover may have lower species abundancy comparing to only planting orchard grass and companion planting.
